# Supplementary material for: Content-rich biological network constructed by mining PubMed abstracts
Source: BMC Bioinformatics. 2004 Oct 8;5:147. doi: 10.1186/1471-2105-5-147 (PMC528731; doi:10.1186/1471-2105-5-147)
Supplement: Additional File 2 — The original results of the above study (non-essential files are deleted to keep the file size under the limit set by BMC bioinformatics). [file 1471-2105-5-147-S2.bz2 › chilibotAdditionalFile2/dip20/26ID8999548E89/html/IFNG_IL18.html]

 


 **IFNG** and **IL18** 
  
Found 772 abstracts in PubMed, retrieved 20.  
 

 What does Google say? 
 PDF only 
| .edu only 

---

**Interactive relationship** (e.g. stimulation, inhibition, etc)

**Stimulatory relationship**- Nai ; ve CD4 T cells differentiate into interferon gamma  [ **IFNG** ]  IFN gamma  [ **IFNG** ]  producing Th1 effector cells when stimulated by interleukin 18  [ **IL18** ]  IL 18  [ **IL18** ]  and IL 12.  Ref: 12829194 Cardiovasc Res< MedlineTA>Cardiovasc Res,
- Naive CD4 CD62L Tcells from normal C57BL 6 mice or from DO11.10 Rag2 OVA specific TCR transgenic mice secreted substantial amounts of IFN gamma  [ **IFNG** ]  when stimulated concurrently with high dose IL 2 plus IL 12 or IL 18  [ **IL18** ] .  Ref: 12811838 Eur J Immunol, 2003
- However, because interleukin IL 18  [ **IL18** ]  acts synergistically with IL 12 to augment Th1 responses and interferon IFN gamma  [ **IFNG** ] , we designed an EGT protocol using IL 12 IL 18  [ **IL18** ]  with the aim of enhancing the antitumor effects obtained with IL 12 alone.  Ref: 12820368 Anticancer Res< MedlineTA>Anticancer Res, 2003
- Our data show that enhanced IFN gamma  [ **IFNG** ]  production in the lungs of bleomycin treated mice is at least partly IL 12 and IL 18  [ **IL18** ]  dependent.  Ref: 12857673 Am J Physiol Lung Cell Mol PhysiolAm J Physiol Lung Cell Mol Physiol, 2003
- IL 18  [ **IL18** ]  is a cytokine structurally and functionally related to IL 1 that, in synergy with IL 12, stimulates the synthesis of IFN gamma  [ **IFNG** ]  from T lymphocytes and natural killer cells.  Ref: 12884303 Eur J ImmunolEur J Immunol, 2003
**Neutral relationship**- IL 12 and IL 18  [ **IL18** ]  activate the synthesis of IFN gamma  [ **IFNG** ]  by innate immune cells, including NK, NKT and gammadeltaT cells and promote the differentiation of Th1 type acquired immune responses.  Ref: 12825993 Rev Iberoam Micol, 2002
- In the gut of non infected piglets, IL 1 beta, IL 8 and IL 18  [ **IL18** ]  were detected whereas TNF alpha and IFN gamma  [ **IFNG** ]  were mostly missing.  Ref: 12879755 Folia Microbiol (Praha), 2003
- CD4 and CD8 T cells induced IL 18  [ **IL18** ]  from DC and primed for IL 12 p70 secretion via interferon gamma  [ **IFNG** ]  IFN gamma  [ **IFNG** ]  or tumour necrosis factor alpha TNF alpha.  Ref: 12871213 Immunology, 2003
- Interleukin 18  [ **IL18** ]  IL 18  [ **IL18** ] , a monokine that shares functional abilities with IL 12, is a potent inductor of interferon gamma  [ **IFNG** ]  IFN gamma  [ **IFNG** ]  by Th1 and natural killer NK cells.  Ref: 12858406 J Med Virol, 2003
- The protective effect of IL 18  [ **IL18** ]  bp Fc was accompanied by modified ex vivo immune responses, in that spleen cells and peritoneal macrophages contained fewer IFN gamma  [ **IFNG** ]  secreting cells and released lower amounts of nitrite an index of nitric oxide production and IL 1beta.  Ref: 12884303 Eur J ImmunolEur J Immunol, 2003
- Anti IL 12 and anti IL 18  [ **IL18** ]  antibodies decreased IFN gamma  [ **IFNG** ]  production by these cells.  Ref: 12857673 Am J Physiol Lung Cell Mol PhysiolAm J Physiol Lung Cell Mol Physiol, 2003
- RESULTS IL 12 IL 18  [ **IL18** ]  EGT inhibited tumor growth significantly better than IL 12 EGT, which was consistent with significantly higher intratumoral levels of IFN gamma  [ **IFNG** ] .  Ref: 12820368 Anticancer Res< MedlineTA>Anticancer Res, 2003

**Non-interactive relationship** (e.g. studied together, co-existance, homology, etc.)

- However, mRNA expression for IL 18  [ **IL18** ]  and interferon gamma  [ **IFNG** ]  IFN gamma  [ **IFNG** ]  was downregulated, and the expression of mRNA for the anti inflammatory cytokine transforming growth factor beta4 TGF beta 4 was upregulated.  Ref: 12859858 J Interferon Cytokine Res, 2003
- Sepsis is initiated and perpetuated by the overzealous systemic production of proinflammatory cytokines such as tumor necrosis factor TNF alpha, interferon IFN gamma  [ **IFNG** ] , interleukin IL 12, and IL 18  [ **IL18** ]  sometimes resulting in excessive tissue injury and death.  Ref: 12874588 Surgery, 2003
- Thus, deformed cortical bone and a decreased turnover rate of lumbar trabecular bone are characteristic of IL 18  [ **IL18** ]  TG mice, and these features might be associated with the increased expression of IFN gamma  [ **IFNG** ]  and IL 18  [ **IL18** ]  in the bone marrow.  Ref: 12817749 J Bone Miner Res, 2003
- Because IFN gamma  [ **IFNG** ]  plays a key pathogenic role in the development of murine immunoinflammatory diabetes induced by multiple low doses of streptozotocin STZ we investigated the effect of negating the actions of endogenous IL 18  [ **IL18** ]  in this model by administering recombinant IL 18  [ **IL18** ]  binding protein Fc IL 18  [ **IL18** ]  bp Fc.  Ref: 12884303 Eur J ImmunolEur J Immunol, 2003
- This study shows that inactivated parapoxvirus ovis Orf virus, strain D1701 PPVO, induces an autoregulatory cytokine response that involves the upregulation of IL 12, IL 18  [ **IL18** ] , IFN gamma  [ **IFNG** ]  and other T helper 1 type cytokines and their subsequent downregulation, which is accompanied by induction of IL 4.  Ref: 12810878 J Gen Virol< MedlineTA>J Gen Virol, 2003
- Deformed cortical bone and decreased turnover rate of lumbar trabecular bone are consistent with increased expression of IFN gamma  [ **IFNG** ]  and IL 18  [ **IL18** ]  in the bone marrow.  Ref: 12817749 J Bone Miner Res, 2003
- We examined the effect of PGE2 on the expression of ICAM 1 and B7 in the human mixed leukocyte reaction MLR in the presence or absence of IL 18  [ **IL18** ] .METHODS We measured the expression of ICAM 1, B7.1, and B7.2 on human monocytes by flow cytometry and determined the associated production of interferon gamma  [ **IFNG** ]  and IL 12 by enzyme linked immunosorbent assay.  Ref: 12829919 Transplantation, 2003
- IL 12, IL 18  [ **IL18** ] , and IFN gamma  [ **IFNG** ]  levels in supernatants of the liver MNCs stimulated with LPS in CLP mice were significantly higher than those in sham mice 7 days after the procedure.  Ref: 12874588 Surgery, 2003
- Responsiveness of CD4 T cells to the IFN gamma  [ **IFNG** ]  inducing cytokines IL 12 and IL 18  [ **IL18** ]  is generally thought to be acquired only after stimulation via the TCR.  Ref: 12811838 Eur J Immunol, 2003
- Recent reports demonstrated that P. acnes treatment promotes IL 12 and IL 18  [ **IL18** ]  synthesis in mice inducing IFN gamma  [ **IFNG** ]  release, enhancement of IgG2a switch and inhibition of Th2 cell expansion.  Ref: 12880687 Immunol Lett, 2003
- Furthermore, serum IL 12 and IL 18  [ **IL18** ]  levels and liver MNCs IL 12, IL 18  [ **IL18** ] , and IFN gamma  [ **IFNG** ]  production were significantly increased in CLP mice compared with sham mice after LPS challenge.  Ref: 12874588 Surgery, 2003
- uNK cells proliferate, produce cytokines interferon gamma  [ **IFNG** ]  IFN gamma  [ **IFNG** ]  and interleukin 18  [ **IL18** ]  IL 18  [ **IL18** ]  and IL 27, and terminally differentiate into granulated lymphocytes.  Ref: 12887272 Reproduction, 2003
- Whereas IL 18  [ **IL18** ]  significantly induced the expression of ICAM 1, B7.1, and B7.2 on monocytes in MLR and the production of interferon gamma  [ **IFNG** ]  and IL 12, PGE2 inhibited these IL 18  [ **IL18** ]  initiated enhancements.  Ref: 12829919 Transplantation, 2003
